# Bone marrow mesenchymal stem cells in rheumatoid arthritis, spondyloarthritis, and ankylosing spondylitis: problems rather than solutions?

**DOI:** 10.1186/s13075-019-2014-8

**Published:** 2019-11-13

**Authors:** Jean-Marie Berthelot, Benoit Le Goff, Yves Maugars

**Affiliations:** Centre Hospitalier Universitaire de Nantes, Nantes, France

**Keywords:** Stem cells, Stromal, Mesenchymal, Bone marrow, Rheumatoid arthritis, Spondyloarthritis, Ankylosing spondylitis, Epigenetic, Synovium, Enthesis

## Abstract

**Background:**

Bone marrow mesenchymal stem cells (BM-MSCs) can dampen inflammation in animal models of inflammatory rheumatisms and human osteoarthritis. They are expected to be a solution for numerous human conditions. However, in rheumatoid arthritis (RA) and spondyloarthritis (SpA), subsets of subchondral BM-MSCs might conversely fuel synovitis and enthesitis.

**Main text:**

Abnormal behaviour of BM-MSCs and/or their progeny has been found in RA and SpA. BM-MSCs also contribute to the ossifying processes observed in ankylosing spondylitis. Some synovial fibroblastic stem cells probably derive from BM-MSCs, but some stem cells can also migrate through the bare zone area of joints, not covered by cartilage, into the synovium. BM-MSCs can also migrate in the synovium over tendons. Sub-populations of bone marrow stem cells also invade the soft tissue side of enthesis via small holes in the bone cortex. The present review aims (1) to make a focus on these two aspects and (2) to put forward the hypothesis that lasting epigenetic changes of some BM-MSCs, induced by transient infections of the bone marrow close to the synovium and/or entheses (i.e. trained immunity of BM-MSCs and/or their progeny), contribute to the pathogenesis of inflammatory rheumatisms. Such hypothesis would fit with (1) the uneven distribution and/or flares of arthritis and enthesitis observed at the individual level in RA and SpA (reminiscent of what is observed following reactive arthritis and/or in Whipple’s disease); (2) the subchondral bone marrow oedema and erosions occurring in many RA patients, in the bare zone area; and (3) the frequent relapses of RA and SpA despite bone marrow transplantation, whereas most BM-MSCs resist graft preconditioning.

**Conclusion:**

Some BM-MSCs might be more the problem than the solution in inflammatory rheumatisms. Subchondral bone marrow BM-MSCs and their progeny trafficking through the bare zone area of joints or holes in the bone cortex of entheses should be thoroughly studied in RA and SpA respectively. This may be done first in animal models. Mini-arthroscopy of joints could also be used in humans to specifically sample tissues close to the bare zone and/or enthesis areas.

## Background section

Stem cells, either haematopoietic or stromal, are multi-potent cells that are self-renewable. Haematopoietic stem cells (HSCs) are maintained in a specialized bone marrow (BM) niche, which consists of osteoblasts, endothelial cells, and a variety of BM mesenchymal stem cells (BM-MSCs) with numerous functions. Some BM-MSCs expressing CD271 and stage-specific embryonic antigen-4 provide a supportive microenvironment for the maintenance of HSCs and haematopoiesis. Most BM-MSCs are capable of differentiating into various cell types (mainly bone, fat, and cartilage). Last, they also exert modulatory effects on cells of both the innate and adaptive immune responses. For instance, some BM-MSCs (CXCL12-positive BM-MSCs) regulate immunological memory by organizing survival niches for plasma cells, and others (CXCL12-negative BM-MSCs) for memory T cells, while keeping T cells quiescent through IL-7 expression [1]. In moderate concentrations, they induce the differentiation of regulatory T cells (Tregs) and maintain their inhibitory functions, as well as the Tregs/Thelpers balance [2].

In osteoarthritis, normal BM-MSCs can contribute to repair damage cartilage [3]. BM-MSCs also dampen the low-grade synovial inflammation associated with this osteoarthritis, as recently confirmed by a clinical trial in humans [4].

**Fig. 1:**
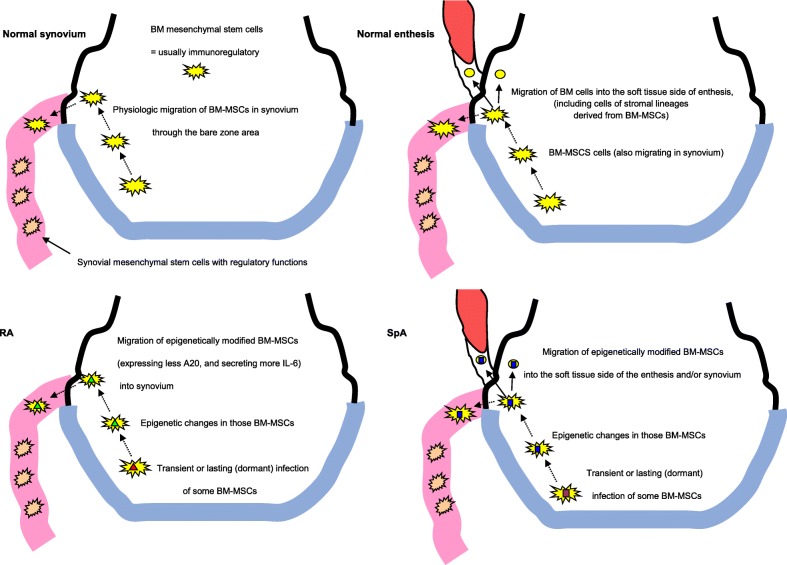
Upper left: in normal joints, most mesenchymal stem cells are resident cells from the synovium, but a small subset of bone marrow mesenchymal stem cells (BM-MSCs) can migrate from the bone into the synovium through canals of the bare zone areas [6]. Upper right: at the synovio-entheseal complexes, populations of BM cells can also invade the soft tissue side of the enthesis via holes in the subchondral bone plate [7]. Lower left: in RA, a small subset of epigenetically modified BM-MSCs from the subchondral bone marrow could migrate into the joint, this migration being enhanced by mechanical stretch [8]. BM-MSCs and their progeny can become pro-inflammatory (with secretion of interferon-gamma [9]), especially outside the bone marrow and when exposed to citrullinated fibrinogen [10]. In RA, those BM-MSCs express less A20 and secrete more IL-6 [11]. Their pro-inflammatory phenotype in RA synovitis could contribute to the typical erosions of this disorder, which occur in the bare zone areas, where BM-MSCs traffic from the bone into the synovium. Lower right: in SpA, the combined treatment of BM-MSC with IL-22, IFN-gamma, and TNF results in increased BM-MSC proliferation and migration [12]. The migration of epigenetically modified BM-MSCs in enthesis could contribute to inflammation, sometimes followed by ossification since BM-MSCs of AS patients have an intrinsic greater potential for osteogenic differentiation [13, 14], further enhanced by IL-22 secretion [12]

BM-MSCs are also of great interest for both the treatment and the understanding of inflammatory rheumatisms, since (1) normal BM-MSCs have been proposed as promising tools for the long-term treatment of rheumatic diseases and several autoimmune diseases [5] and (2) conversely, abnormal behaviour of some BM-MSCs and/or their progeny has been found in rheumatoid arthritis (RA) and ankylosing spondylitis (AS) or spondyloarthritis (SpA). They could locally contribute to synovitis and enthesitis. Indeed, some synovial fibroblastic stem cells probably derive from BM-MSCs, and some BM stem cells (as well as synovial nurse-like cells) can migrate through the bare zone area of the joint (where the synovium directly overlies the bone) into the synovium (Fig. 1). Sub-populations of BM stem cells can also invade the soft tissue side of enthesis via small holes in the bone cortex (Fig. 1).

In other words, whereas grafts of normal autologous or allogeneic BM-MCS might become one of the best solutions to treat various inflammatory rheumatisms, a small subset of subchondral BM-MSCs could on the opposite be an underrated issue. Indeed, acquired defects of such cells might fuel RA and SpAs, as deduced since 1990 from experiences of BM transplantation [15].

The present narrative review aims to make a focus on these aspects and to put forward the hypothesis that trained immunity [16] of some BM-MSCs (induced by transient trafficking of dead or alive pathogens in subchondral BM close to joint and/or entheses [17]) contributes to the pathogenesis of inflammatory rheumatisms (Fig. 1).

## Results

### Some synovial fibroblast stem cells and related cells (like nurse-like cells) have a BM origin and can migrate from BM to joint and enthesis

RA synovitis is not only the consequence of over-activation of haematopoietic cells in the synovium: RA synovitis is also associated with an aberrant joint fibroblast activation and proliferation, which contributes to joint destruction. This fibroblast proliferation depends on resident fibroblasts and synovial mesenchymal stem cells (MSCs), a heterogenous population mostly of synovial origin. However, synovial MSCs can also derive from pericytes, endothelial cells, adipose tissue, ligaments, menisci, and a small subset of BM-MSCs [18]: some multi-potent BM-MSCs have indeed been found in the synovial membrane on several occasions [19, 20] (Fig. 1). Those multipotential MSCs from the synovial fluid and BM of patients with inflammatory arthritis were reported to be negative for CD45, but positive for D7-FIB, CD13, CD105, CD55, and CD10 [20]. BM-MSCs were first thought to be brought into the synovium via blood vessels of the systemic circulation. Indeed, in a murine model of osteoarthritis, when cultured rheumatoid synovial fibroblasts with human cartilage were implanted subcutaneously into severe combined immunodeficient mice, they migrated into the synovial tissues of the distant osteoarthritic joints [21].

However, at least a subset of BM-MSCs could also migrate more directly from subchondral BM into the adjacent synovium (Fig. 1).

First, some studies employing lineage tracing and transgenic Lewis rats demonstrated that fibroblastic stromal cells (FSCs) of the BM (labelled with a fluorescent probe or 3HTdr) examined before and after induction of polyarthritis were found to migrate from BM into the joint cavity through canals observed in the bare zone of the joint [6]. Those cells then proliferated in the synovial tissue [6]. Although normal BM-MSC cells physiologically migrate from BM to the synovium to locally dampen excessive immune response (Fig. 1), some pathophysiological cells of RA synovitis might similarly migrate from BM into the adjacent joint space and play roles in inflammation or tissue damage (Fig. 1). This BM contribution to synovial hyperplasia has been recently confirmed in a model of joint surface injury [22]. The majority of BM-derived cells in the synovium were haematopoietic, but a minority did express the pan-fibroblast/MSC marker Pdgfrα [22]. Although the influx of BM-derived cells should not be responsible for all the accumulation of fibroblastic synoviocytes seen in murine models of RA (mostly linked to local fibroblast proliferation) [16], migration of a small subset of modified BM-MSCs from BM into the joint could be sufficient to induce long-lasting changes in the homeostasis of the synovium or enthesis. Even in low numbers, BM-MSCs could indeed contribute to synovitis and/or enthesis through the release of extracellular vesicles (EVs) (exosomes, microparticles, and apoptotic bodies) [23]. Indeed, EVs reproduce the main functions of the parental cells [23].

Second, the migration of other BM-stromal cells towards RA synovium (nurse-like cells (NLCs)) has also been demonstrated. Among fibroblastic stromal cells in synovial tissue and BM, NLCs are a unique population having the specific capacity to promote pseudo-emperipolesis (adhesion and holding beneath) of lymphocytes and their growth. Therefore, nurse-like cells probably contribute to the immunopathogenesis of RA and to the localization of inflammation within specific joints [24, 25]. Indeed, the survival niches promoted by RA-NLCs, either in BM or in synovium, appear to play important roles in supporting immunological functions in RA BM and synovial tissues [24].

At the synovio-entheseal complexes, populations of BM cells can also invade the soft tissue side of the enthesis via holes in the subchondral bone plate [7] (Fig. 1). Indeed, 96% of entheses have small holes in the cortical shell (typically 100–400 μm wide) [7], whereas the size of MSCs ranges from 17 to 31 μm. The most common source of inflammatory cells seen at the entheses (judging from their anatomic proximity) is likely to be BM [26] (although some may also derive from blood vessels in the endotenon or endoligament [26], and/or periosteal stem cells).

Migration of BM-MSCs into peripheral tissues with differentiation in fibroblastic cells is not restricted to the joint synovium or enthesis, since BM-MSCs can also migrate in the synovium over tendons [27]. It has also been shown ex vivo an increase migration of BM-derived MSCs transplanted in the skin, which was accelerated following mechanical stretch [8]. Mechanical stretch indeed upregulates SDF-1alpha and recruits circulating BM-MSCs through the SDF-1α/CXCR4 pathway [8]. This process is increased by IL-3: in vitro, IL-3 upregulates the expression of chemokine receptor 4 (CXCR4) on BM-MSCs, significantly enhancing migration and motility of BM-MSCs towards SDF-1α [28].

As SDF-1 is strongly expressed in the synovium [8] and bursitis, its upregulation during RA synovitis or bursitis could similarly recruit BM-MSCs in synovia or bursae through the SDF-1α/CXCR4 pathway [8]. Indeed, in RA, SDF-1 and CXCR4 in the synovium are also associated with the disease activity and bone joint destruction [29]. In a model of anterior cruciate ligament rupture, enhanced migration of BM-MSCs at the myotendinous junction (enthesis) of the quadriceps was also associated with increased SDF-1α immunostaining.

### Abnormal behaviour of BM-MSCs and/or their progeny has been found in RA, SpA, and AS

In normal joints, the role of MSCs, including BM-MSCs, is beneficial, through the maintenance of joint homeostasis and repair of small lesions. Conversely, some subsets of MSCs, and BM-MSCs, might be defective, and even deleterious, in chronic inflammatory rheumatisms.

This has been first ascribed to focal inflammation, since, in co-culture of synovium-derived MSCs and T cells from RA patients, the repair function of BM-MCS and MSCs is repressed by the inflammatory milieu (differentiation of BM-MCSs is blocked in RA patients) [30]. Tumour necrosis factor alpha (TNF-α) indeed prevents the mesenchymal differentiation capabilities of MSCs [31]. A negative correlation between synovial bone marrow MSCs’ chondrogenic and clonogenic capacities and the magnitude of synovitis in RA patients has also been observed [31]. Inflammation similarly partly accounts for the inhibition of adipogenesis of MSCs. This contributes to the bone oedema observed in RA subchondral BM and in BM of SpA entheses.

Usually, BM-MSCs are immunosuppressive and can inhibit neutrophil recruitment to TNF-α-treated endothelial cells. Conversely, BM-MSC-derived adipocytes in inflamed tissues are no longer able to suppress neutrophil adhesion [32]. An increase in proliferation of T cells was also observed when IL-17A and TNF-α were added, alone or in combination, to co-culture of synovium-derived MSCs and T cells from RA patients [33]. Nevertheless, pre-treatment of human MSCs with inflammatory factors only influences the expression of migration and adhesion receptors of MSCs, but it does not reduce their migration to cartilage or synovium (in vitro) or adhesion (in vivo). Similarly, TNF-α does not alter MHC class II expression by BM-MSCs, and, if interferon-gamma (IFN-gamma) priming induced an upregulation of MHC expression, co-stimulatory molecule expression was not upregulated [34].

Therefore, other explanations than a lower immunomodulatory activity of mesenchymal stem cells (MSCs) in an inflammatory environment should also be considered to account for the worsening role of BM-MSCs in animal models of chronic inflammatory rheumatisms. Indeed, in collagen-induced arthritis (CIA), BM-MSCs did not hamper the development of arthritis and rather accelerated it through enhancement of IL-6 production [35].

As several stem cell differentiation appears disrupted in human inflammatory rheumatic diseases [5], some modified BM-MSCs or their progeny could directly worsen arthritis. This could be even more important if some fibroblast-like synoviocytes undergoing proliferation represent a functional stage of former BM-MSCs [36]. There are already evidences that in environments other than BM some BM-MSCs can have pro-inflammatory effect, at least for enhancing subsets of T cells.

#### In RA

For instance, in a co-culture of peripheral blood mononuclear cells from healthy blood donors with BM-MSCs of healthy donors, the interaction of peripheral blood mononuclear cells (PBMCs) with BM-MSCs inhibited Th1 and Th2 responses, but promoted Th17 cell expansion, as early as 24 h [9]. This was also observed when culturing PBMCs with fibroblast lining cells (FLS) from RA patients [9]. This increase required both cell–cell contact and soluble factors and was ascribed to expansion of memory T cells. This has been confirmed by the observation that memory, but not naive, Th17 cells can enhance their IL-17A production in the presence of stromal cells [37]. Usually, IFN-gamma negatively regulates the development of Th17 cells when added to lymphocyte cultures alone [9]. However, in an equine model, IFN-gamma increased BM-MSCs [35] so that IFN-gamma, through the stimulation of BM-MSCs in other places than BM, might indirectly have a positive role on IL-17A production.

Excessive migration of BM-MSCs in the synovium or enthesis combined with local inflammation could therefore fuel excessive immune responses (especially if IFN-gamma is secreted). In humans, whereas OA fibroblast-like synoviocytes or RA fibroblast-like synoviocytes similarly enhanced IL-17A and IL-6 production, only RA fibroblast-like synoviocytes enhanced IFN-gamma production [9].

Nevertheless, the ectopic presence of BM-MSCs in the synovium is probably not sufficient to induce long-lasting immune responses, and some acquired intrinsic changes in BM-MSCs within the synovium could be required to sustain chronic synovitis of RA. Several evidences suggest that some BM-MSCs might behave abnormally and/or are epigenetically modified in RA, following exogenous triggers, or metabolic changes. Those changes probably occur first in the subchondral BM niches (which could account for the possible occurrence of subchondral bone marrow oedema without synovitis in some very early RA).

First, when exposed to citrullinated fibrinogen (the auto-antigen targeted the most specific RA auto-antibodies), BM-MSCs increased their production of IL-6, IL-8, and the chemokine CCL2 [10]. Citrullinated fibrinogen also impaired immunomodulatory function of BM-MSCs and reduced their production of the key immunomodulatory molecule indoleamine 2,3-dioxygenase, by triggering toll-like receptors (TLRs) [10].

Second, comparisons of synovium-derived MSC of RA and controls showed that 3117 genes were upregulated and 1711 genes were downregulated in RA-MSCs [38]. Genes of the mitogen-activated protein kinases (MAPK) signalling and RA pathways were upregulated, and those of the p53 signalling pathway were downregulated [38]. Other studies confirmed that 523 low-methylated regions of DNA were specific to RA synoviocytes, as compared with osteoarthritis patients [39].

Third, it was recently shown that A20 is decreased in BM-MSCs of RA patients [11]. A20 (product of the TNFAIP3 gene) is highly expressed in many cell types after the stimulation of TNF-α, to prevent excessive activation of NF-kB following exposure of the cells to TNF-α. As the lack of A20 expression in RA BM-MSCs is associated with more IL-6 secretion [11], and dysregulation of the Th17/Treg balance, this acquired defect of RA BM-MSCs could contribute to arthritis pathogenesis (Fig. 1).

#### In SpA and AS

In mice defective for A20 expression in myeloid cells, enthesitis was also found to be an early inflammatory lesion. A20 negatively modulated signal transducer and activator of transcription 1 (STAT1)-dependent gene transcription in myeloid cells [40]. In vivo inhibition of the Jak-STAT1 pathways resulted in a significant reduction of enthesitis, both clinically and histopathologically [40]. Whether a similar defect in BM-MSCs could also contribute to enthesis following TNF stimulation in human is not proven yet, but would not be surprising.

Indeed, using an established TNF transgenic murine model (which develops a SpA-like disease characterized by peripheral joint arthritis, sacroiliitis, enthesitis, and Crohn’s-like inflammatory bowel disease), it was shown that selective TNFRI expression in mesenchymal cells resulted in a fully arthritic–spondyloarthritic and intestinal phenotype [41]. This indicates that mesenchymal cells are primary and sufficient targets of TNF in these pathologies [41]. Indeed, enthesitis occurred equally in the presence or absence of mature T and B cells, underscoring that only mesenchymal cells were important [42]. Similarly, BM grafting experiments demonstrated that the development of arthritis/enthesitis just requires TNF receptor I (TNFRI) expression in the radiation-resistant compartment [41]. Those radiation-resistant cells are known to be a sufficient target of TNF in the development of Crohn’s-like bowel disease [41]. Notably, overexpression of TNF by MSCs was associated with early activation of synovial fibroblasts [41]. This would fit with either a filiation between BM-MSCs and synovial fibroblast or a control of the latter by the former.

As MSCs from BM of enthesis are difficult to obtain, most studies performed so far on BM-MSCs from AS or SpA patients used either BM-MSCs from remote sites (i.e. sternum) or induced pluripotent stem cells (for instance, from dermal fibroblasts).

In patients with active AS, the BM-MSCs (from sternal BM) showed normal proliferation, cell viability, surface markers, and multiple differentiation characteristics. However, a significantly reduced immunomodulation potential (decreased 68 ± 14%) of those BM-MSCs was also observed (the frequencies of Treg and Fox-P3+ cells decreased, while CCR4+CCR6+ Th cells increased) [43]. Other works also concluded that MSCs from AS patients have lower immunoregulatory abilities. Indeed, as they secrete more monocyte chemoattractant protein 1 than healthy donors, monocyte migration ensues following BM-MSC activation (as well as macrophage polarization, and enhanced TNF-α secretion) [44].

In psoriasis, BM-MSCs also exhibit abnormal cytokine secretion [45].

As reactive arthritis sometimes antedates SpA, the hypothesis that transient or lasting silent infection of BM niches could contribute to modify the epigenetics of BM-MSCs in inflammatory rheumatisms is attractive (Fig. 1). Indeed, it has been shown that bacteria like *Mycobacterium tuberculosis* and several viruses can remain alive in human BM-MCSs for very long periods [17]. It has not yet been proven that bacteria or their antigens modify BM-MSC behaviour in patients with reactive arthritis who later develop SpA, but some clues might fit with this possibility. For instance, the observation of an elevated TNF receptor-associated factor 4 (TRAF4) expression in MSCs from AS patients impairs lipopolysaccharides (LPS)-induced autophagy [46]. This could indeed promote transient infections in the bone marrow niches of enthesis [17], and/or further trained immunity [16] of transiently infected BM-MSCs.

An alternative hypothesis to explain the involvement of BM-MSCs in SpA pathogenesis could be the secretion of IL-22 by various cells in entheses. Indeed, IL-22 enhances proliferation and migration of BM-MSCs in enthesis, provided that other cytokines are present. Combined treatment of BM-MSC with IL-22, IFN-gamma, and TNF results in increased MSC proliferation and migration, which is not seen in cells treated with IL-22 alone [12] (Fig. 1).

Most studies focusing on the contribution of BM-MSCs to the pathogenesis of SpA and AS addressed their involvement in the ossifying process of the entheses, characteristic of long-lasting AS. This is highly probable, as high tensile loads promote osteogenic differentiation, whereas diffuse hyperostosis (which can mimic AS) is also driven by over-activation of BM-MSCs.

In AS, some works concluded that the ossification of entheses might result from an enhancement of BM-MSC osteogenesis following IL-22 exposure alone (Fig. 1). Indeed, a combination of IFN-gamma and TNF with or without IL-22 rather suppressed it [12]. This sequence could account for the observation that ossification of entheses often occurs following clinical flares, and have not been much impaired by long-term treatment with anti-TNF drugs. Other works concluded that BM-MSCs of AS patients had already an intrinsic greater potential for osteogenic differentiation, as compared with BM-MSCs of healthy donors [13]. A study of the osteogenic differentiation capacity of sternal BM-MSCs from AS, as compared with healthy donors, indeed demonstrated an imbalance between more BMP-2 (bone morphogenic protein-2) and less Noggin secretion, which was associated with osteogenic differentiation of AS-MSCs [13]. BMP2 expression in BM-MSCs of ossifying entheses was even higher in AS patients [14]. The dysfunction resulting from this BMP2 overexpression finally led to enhanced osteogenic differentiation [14]. BM-MSCs of patients with AS also inhibit too much osteoclastogenesis through the miR-4284/CXCL5 axis, a property which combines with their stronger osteogenic differentiation. Last, a study of AS-MSCs and healthy donor MSCs induced with osteogenic differentiation medium for ten days showed that four long noncoding RNA (lnc) were overexpressed in AS-MSCs and associated with increased osteogenesis, including lnc-ZNF354A-1, lnc-LIN54-1, lnc-FRG2C-3, and lnc-USP50-2 [47].

### Some clinical observations would fit with the hypothesis of creeping provocations of BM-MSCs at the onset of RA and SpA

Those epigenetic changes in BM-MSCs might be the long-term consequences of transient or repeated trafficking in SpA and RA BM of antigens from pathogenic gram-negative bacteria or bacteria from microbiota (Fig. 1) [48].

First, whereas palindromic rheumatisms sometimes antedate RA or SpA, the same holds true for Whipple’s disease (a latent infection of gut and other tissues, including synovium, by the bacteria *Tropheryma whipplei*). Therefore, remittent infections by slow growing pathogens or gut synbionts, with transient migration in joints and/or subchondral BM, could also contribute to the palindromic onset of some RA or SpA [48].

Second, distribution of inflammation and erosive disease is often confined to some joints, bursae, tendons, and/or enthesis (which differ according to patients). A major role of mechanical strain on BM-MSCs (rather than cells of the adaptative immunity) to determine the site-specific localization of inflammation has been promoted [49]. Indeed, mechano-stimulation of MSC cells induces CXCL1 and CCL2 for the recruitment of classical monocytes, which can differentiate into bone-resorbing osteoclasts [49] (whereas phenotype of adaptive cells is not altered by this mechano-stimulation). Similarly, in the murine model of enthesitis and arthritis dependent on stromal cell overexpression of TNF (TNF(ΔARE)), hind limb unloading of mice significantly suppressed inflammation of the Achilles tendon, as well as further ossification [42].

However, the striking variations/flares of disease activity of SpA (and at a lesser degree in RA) observed during prospective studies of individual patients cannot be only explained by more intense mechanical strains. Patients usually deny any relationship between those flares and their activity. Similarly, the uneven distribution of enthesis and/or arthritis (which can seriously affect one finger and not its neighbour) does not fit well with the mechanical strain hypothesis. The uneven distribution of arthritis/enthesitis would be better explained by a random metastatic spreading of some pathogen(s) towards several BM niches at the very onset of inflammatory disorders. Flares could be better explained by re-exposure of the immune system to those pathogen(s). A spreading from the colonic mucosa to the bones, first through the gut, then the sacro-iliac and paraspinal lymphatics, could also contribute to account for the association of reactive arthritis, SpA, and AS with sacroiliitis and spinal involvement [50]. Such lymphatic trafficking has indeed been demonstrated for *Tropheryma whipplei* in Whipple’s disease, which can mimic SpA, as well as RA.

Whipple’s disease followed by SpA or RA would therefore be one of the best human models to test the hypothesis that repeated bacterial translocation into subchondral BM niches can induce very long-lasting epigenetic changes in BM-MSCs of genetically predisposed hosts. Such ‘trained immunity’ of long-live BM-MSCs might sustain low-grade arthritis or enthesitis for years following silent and transient BM infection. Epigenetic changes in some BM-MSCs could persist despite the disappearance of bacteria (including the killing of replicating following antibiotic treatment), as also observed in ‘reactive arthritis’.

Intracellular bacterial (or viral) infections of BM-MSCs themselves should not to be ruled out as a trigger for such lasting trained immunity. MSCs possess anti-bacterial characteristics and are equipped with pattern recognition receptors, including TLRs. However, it could be demonstrated in canine MSCs that many gastro-intestinal pathogens and probiotic bacteria (including *Salmonella*, *Escherichia* *coli*, *Listeria*, *Lactococcus*, *Lactobacillus*, and *Bifidobacterium*) adhere to, and invade MSCs, at least in vitro, leading to increased secretion of IL6, IL8, and prostaglandin-E2 (PGE2) by MSCs. Importantly, and surprisingly, none of the bacteria induced MSC death or apoptosis (as seen with epithelial association) [51]. Those findings fit with the recent demonstration that several bacteria can remain alive for very long periods within human BM-MSCs (including *Mycobacterium tuberculosis* [17]).

It should be stressed that MSCs from different tissue sources (including BM-MSCs) and different species may have variable specific responses to microbial interaction. Indeed, mesenchymal stem/progenitor cell properties can differ according to animal and human tissues. For instance, a recent study comparing the biological activity of MSCs isolated from the BM, adipose tissue, skeletal muscles, and skin showed that long-term culture affected the biological activity of MSCs obtained from those various tissues (their biological activity and some markers differed) [52]. However, BM-MSCs and adipose tissue MSCs revealed similarities in phenotype maintenance, capacity for multi-lineage differentiation, and secretion of bioactive factors, making adipose tissue MSCs an alternative source for BM-MSCs for regenerative medicine and/or other human conditions [52]. Reciprocally, those similarities could make adipose tissue MSCs and their progeny more permissive for the same creeping infections as those observed in vitro or in vivo in human BM-MSCs. For instance, *Mycobacterium tuberculosis* could also be found in human adipose tissues, and it was also shown that *M. tuberculosis* can enter within adipocytes and survives within those cells in a non-replicating state insensitive to the major anti-mycobacterial drugs [53].

Further studies, first performed on animal models of RA and SpA at their very onset, could tell whether transient and silent infections of subchondral BM niches (including by bacteria of the microbiota) might be sufficient to foster the migration of BM-MSCs in adjacent synovium/enthesis. Those studies could also search for epigenetically induced lasting trained immunity [54] of those cells, and their progeny, in synovium or enthesis (Fig. 1). It would also be worth to search for a link between such events and pain, since epigenetic changes in BM-MSCs might also impair the ability of normal BM-MSCs to inhibit neuropathic pain, thus accounting to some ‘secondary fibromyalgia’ associated with RA and SpA.

## Conclusion

Whereas normal BM-MSCs are expected to be a solution for various human disorders, in RA and SpA/AS, a small subset of BM-MSCs might rather be a problem. Studies of those stem cells in synovium, but also subchondral BM and enthesis BM, should be strongly encouraged, first in animal models of RA and SpA. In humans, mini-arthroscopy of joints could be used to try and more specifically sample tissues from the bare zone and enthesis areas where some stem cells traffic from BM into joints and enthesis, respectively.

## Data Availability

Not applicable

## Abbreviations

AS: Ankylosing spondylitis
BM: Bone marrow
BM-MSCs: Bone marrow mesenchymal stem cells
BMP-2: Bone morphogenic protein-2
CIA: Collagen-induced arthritis
CXCR4: Chemokine (C-X-C motif) receptor 4
EVs: Extracellular vesicles
FLS: Fibroblast lining cells
FSCs: Fibroblastic stromal cells
HSCs: Haematopoietic stem cells
lncs: Long noncoding RNAs
LPS: Lipopolysaccharides
MAPK: Mitogen-activated protein kinases
MSCs: Mesenchymal stem cells
NLCs: Nurse-like cells
PBMCs: Peripheral blood mononuclear cells
PGE2: Prostaglandin-E2
Tregs: Regulatory T cells
RA: Rheumatoid arthritis
SDF-1alpha: Stromal derived factor-1alpha
SpA: Spondyloarthritis
STAT1: Signal transducer and activator of transcription 1
TLRs: Toll-like receptors
TNF: Tumour necrosis factor
TRAF4: TNF receptor-associated factor 4
